# Inhibition of NHE1 Overcomes Temozolomide‐Resistance in Glioblastoma via ROS‐AKT/ERK Axis‐Mediated Autophagy Suppression

**DOI:** 10.1002/jbt.71110

**Published:** 2026-09-14

**Authors:** Dan Li, Yuhui Li, Xuan Zheng, Zhuo Wang, Yang Wang, Jun Zhang, Jianxiong Guo, Lei Wang, Jingwu Li, Yongliang Liu, Yufeng Li

**Affiliations:** ^1^ Hebei Key Laboratory of Molecular Oncology Tangshan People's Hospital Hebei China; ^2^ Tangshan Key Laboratory of Cancer Prevention and Therapy Tangshan People's Hospital Hebei China; ^3^ The Cancer Institute Tangshan People's Hospital Hebei China; ^4^ Department of Neurosurgery Tangshan People's Hospital Tangshan Hebei China; ^5^ Department of Pathology Tangshan People's Hospital Hebei China; ^6^ Department of Gastrointestinal Surgery Tangshan People's Hospital Hebei China

**Keywords:** autophagy, glioblastoma, NHE1, ROS‐AKT/ERK axis, temozolomide resistance

## Abstract

Glioblastoma (GBM) is the most aggressive primary brain tumor in adults, with temozolomide (TMZ) resistance being a major barrier to improved clinical outcomes. Dysregulated autophagy is closely associated with TMZ resistance in GBM, but the role of sodium/hydrogen exchanger 1 (NHE1)—a key regulator of intracellular pH homeostasis—in this process remains uncharacterized. We established a TMZ‐resistant GBM cell line (U251TR) and observed that U251TR cells displayed higher autophagic activity, increased reactive oxygen species (ROS) levels, and reduced apoptotic rates compared to parental U251 cells. Treatment with the NHE1‐specific inhibitor HOE‐642 dose‐dependently suppressed cell viability, inhibited NHE1 activity, reduced intracellular ROS production, and promoted apoptosis in U251TR cells, with a positive feedback loop identified between NHE1 and ROS. Mechanistically, HOE‐642 blocked autophagic initiation and flux primarily via the ROS‐AKT/ERK signaling axis, in which reduced ROS downregulated p‐AKT and p‐ERK1/2, where ERK exerted pro‐autophagic effects and AKT exerted anti‐autophagic effects. In vivo, combined HOE‐642 and TMZ treatment significantly reduced xenograft tumor volume and weight, inhibited autophagy, and activated apoptosis compared to TMZ monotherapy, without causing evident systemic toxicity. Our findings identify a novel NHE1‐ROS‐AKT/ERK‐autophagy regulatory axis in TMZ‐resistant GBM, validating NHE1 as a potential therapeutic target to enhance TMZ sensitivity by disrupting the autophagy‐apoptosis balance, and providing a new strategy for GBM treatment.

## Graphical abstract

NHE1 inhibition via HOE‐642 suppresses autophagy through the ROS‐AKT/ERK axis, reversing temozolomide resistance in glioblastoma by disrupting the autophagy‐apoptosis balance and enhancing TMZ sensitivity in vitro and in vivo.

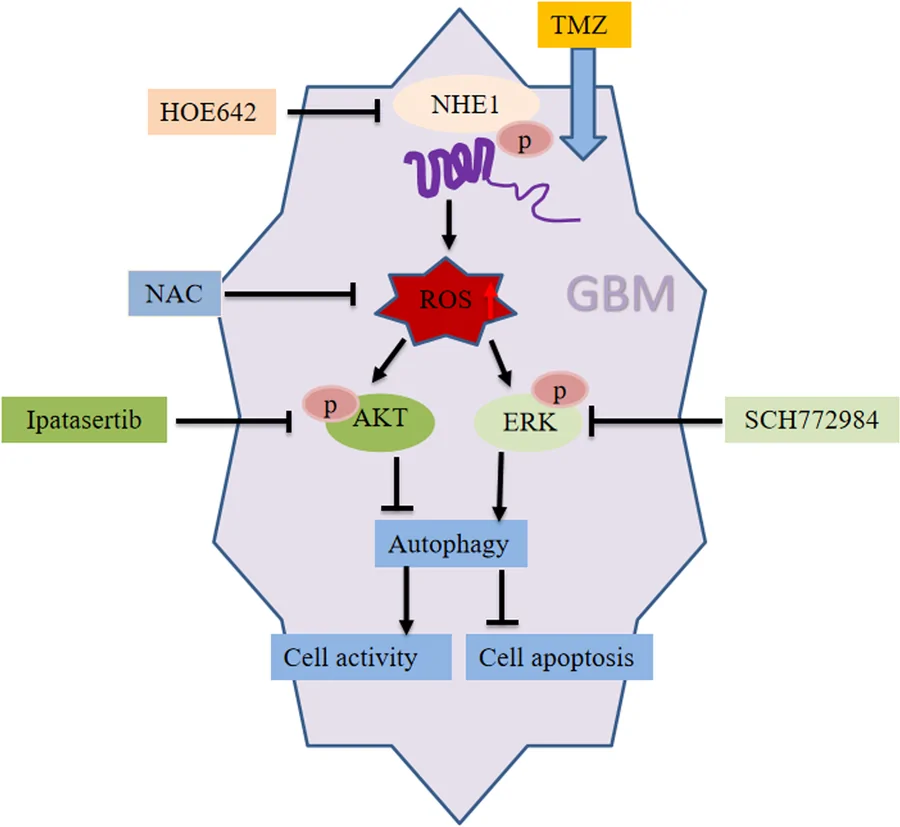

**Abbreviations**
AKT: protein Kinase B
AMPKα: AMP‐activated protein kinase α
BCECF‐AM: 2′,7′‐Bis‐(2‐Carboxyethyl)‐5‐(and‐6)‐Carboxyfluorescein, Acetoxymethyl Ester
CCK‐8: cell counting kit‐8
DAPI: 4′,6‐Diamidino‐2‐Phenylindole
DCFH‐DA: 2′,7′‐Dichlorodihydrofluorescein diacetate
DMEM/F12: Dulbecco's Modified Eagle Medium/Nutrient Mixture F12
ERK: extracellular regulated protein kinases
FACS: fluorescence activated cell sorting
FBS: fetal bovine serum
FIR: fluorescence intensity ratio
GBM: glioblastoma
HOE‐642: NHE1‐specific inhibitor
Ipatasertib: AKT inhibitor
mTOR: mammalian target of rapamycin
NAC: N‐Acetylcysteine
NHE1: sodium/hydrogen exchanger 1
OD: optical density
PBS: phosphate‐buffered saline
PDX: patient‐derived xenograft
pHi: intracellular pH
PI3K: phosphatidylinositol 3‐kinase
PVDF: polyvinylidene fluoride
RAPA: rapamycin
RIPA: Radioimmunoprecipitation Assay
ROS: reactive oxygen species
SCH772984: ERK inhibitor
SD: standard deviation
TEM: transmission electron microscopy
TMZ: temozolomide
U251TR: temozolomide‐resistant U251

## 1 Introduction

Glioblastoma (GBM) is the most prevalent and aggressive primary brain tumor in adults. High invasiveness and limited therapeutic efficacy make it difficult to treat clinically. Recent studies have consistently emphasized the dismal prognosis of GBM with an abysmally low 5‐year survival rate [1, 2, 3]. Tumor recurrence is rampant after initial treatment, largely driven by chemoresistance [4, 5]. The current standard of care for GBM calls for maximal safe surgical resection, followed by radiotherapy and adjuvant temozolomide (TMZ) chemotherapy [6, 7]. However, this multimodal treatment strategy is frequently undermined by TMZ‐specific resistance [8, 9], which accelerates tumor recurrence and drastically worsens patient survival outcomes. Thus, clarifying the molecular mechanisms of TMZ resistance and identifying practical therapeutic targets is essential to develop effective strategies to improve patient prognosis in GBM treatment.

Autophagy is a highly conserved catabolic process mediating the sequestration, degradation, and recycling of cellular components via lysosomal hydrolysis, which is indispensable for maintaining cellular bioenergetic balance and proteostasis under stress [10, 11, 12]. In cancer biology, autophagy plays a dual role: it can either promote cell survival or induce cell death, depending on the cellular context and stress intensity [13, 14]. In GBM, autophagy often acts as a survival mechanism for tumor cells under nutrient scarcity and chemotherapy, providing essential nutrients for cell maintenance [15, 16], while excessive activation induces cell death [17, 18]. Dysregulated autophagy is closely implicated in GBM drug resistance and constitutes a promising therapeutic target.

Sodium/hydrogen exchanger 1 (NHE1), a member of the SLC9A family, is a key regulator of intracellular pH (pHi) homeostasis and cell volume [19]. In tumor cells, NHE1 is frequently overexpressed or hyperactivated, which drives the formation of an acidic extracellular microenvironment and an alkaline intracellular environment. This pH dysregulation promotes tumor proliferation, invasion, and drug resistance [20, 21]. In BRAF V600E mutant GBM cells, NHE1 and ERK phosphorylation form a positive feedback loop, and this circuit drives tumor cell proliferation and invasion [22]. Recent findings reveal that NHE1 modulates autophagy in cancer cells [23]. For instance, curcumin combined with glucose restriction inhibits NHE1 in hepatocellular carcinoma cells [23]. This treatment lowers pHi, decreases ATP and lactate production, and promotes autophagy. However, the role of NHE1 in regulating autophagy in TMZ‐resistant GBM cells and the underlying molecular mechanisms remain largely unexplored.

Reactive oxygen species (ROS) are key mediators of oxidative stress and critical regulators of cellular signaling in GBM [24]. Dysregulated ROS homeostasis with excessive ROS accumulation is strongly linked to GBM progression, TMZ resistance, and autophagy modulation [25, 26]. In cancer cells, ROS modulate NHE1 activity, and NHE1‐mediated pHi regulation in turn alters ROS levels; this reciprocal crosstalk facilitates tumor cell survival, proliferation, invasion, and metastasis [27, 28]. ROS serve as upstream signaling molecules governing autophagic flux: moderate ROS promote protective autophagy, while excessive ROS induce autophagic cell death [29, 30, 31]. This balance strongly influences the response of GBM to TMZ [32]. Despite these connections, how NHE1 regulates ROS homeostasis in TMZ‐resistant GBM and whether the NHE1‐ROS axis mediates autophagy‐dependent TMZ resistance remain unclear.

This study aimed to fill this research gap by investigating the regulatory effects of NHE1 on autophagy in TMZ‐resistant GBM cells and elucidating the underlying molecular mechanisms. We further sought to determine whether NHE1 inhibition modulates autophagic flux by remodeling ROS homeostasis and downstream signaling cascades, as well as to validate NHE1 as a potential therapeutic target for overcoming TMZ resistance.

## 2 Materials and Methods

### 2.1 Cell Culture

Human GBM cell line U251 was purchased from Saier Biotechnology. The drug‐resistant cell line U251TR was established by our research group. The U251 and U251TR cells were cultured in DMEM/F12 medium (GIBCO) supplemented with 10% fetal bovine serum (GIBCO), 100 IU/mL of penicillin, and 100 μg/mL of streptomycin (GIBCO), and incubated at 37°C in an atmosphere containing 5% CO_2_.

### 2.2 Western Blot

Total protein was collected using protein lysis buffer containing 1 µL protease inhibitor (Solarbio) and 1 mL radioimmunoprecipitation assay (RIPA) lysis buffer. The protein concentration was calculated using the BCA assay (Merck). After protein samples were boiled at 99°C for 5 min, the samples were transferred to PVDF membranes (Millipore). Subsequently, the membranes were incubated for 1 h in blocking buffer, then incubated with the primary antibodies overnight at 4°C, followed by second antibodies treatment. The antibodies were used as follows: Anti‐LC3 antibody (Saierbio, Tianjin, China; SRP15376; 1:1000), Anti‐Beclin1 antibody (Saierbio, Tianjin, China; SRP14604; 1:1000), Anti‐P62 antibody (Saierbio, Tianjin, China; SRP11189; 1:1000), Anti‐Caspase 8 antibody (Saierbio, Tianjin, China; SRP14705; 1:1000), Anti‐Caspase 9 antibody (Saierbio, Tianjin, China; SRP12193; 1:1000), Anti‐Caspase 10 antibody (Saierbio, Tianjin, China; SRP10345; 1:1000), Anti‐ERK1/2 antibody (CST, America; 4695S; 1:2000), Anti‐Phospho‐ERK1/2 antibody (CST, America; 9101S; 1:2000), Anti‐AMPKα antibody CST, America; 2532S; 1:2000), Anti‐Phospho‐AMPKα antibody (CST, America; 2535 T; 1:2000), Anti‐mTOR antibody (CST, America; 2983 T; 1:2000), Anti‐Phospho‐mTOR antibody (CST, America; 5536 T; 1:2000), Anti‐Phospho‐AKT Antibody (CST, America; 13038 T; 1:2000), Anti‐AKT Antibody (CST, America; 60203‐2‐Ig; 1:2000), Anti‐NHE1 antibody (proteintech, America; 29761‐1‐AP; 1:2000), Anti‐GAPDH antibody (Saierbio, Tianjin, China; SRP00849; 1:1000). The immunoreactive bands were detected using the ChampGel automatic gel imaging analyzer (SageCreation). Optical band density was quantified using Image J.

### 2.3 Flow Cytometry Analysis

Cell apoptosis determination and cellular ROS production were measured with flow cytometry. All cells under various experimental conditions in six‐well plates were harvested with 0.25% trypsin. Apoptosis was determined by the Annexin V‐FITC apoptosis detection kit (Beyotime, Shanghai, China) following the manufacturer'sprotocol with FACS cytometry (BD FACSAria II; BD Biosciences, San Jose, CA, USA). For ROS measurement, U251/U251TR cells were incubated with DMEM containing 10 μM 2′‐7′‐Dichlorodihydro‐fluorescein diacetate (DCFH‐DA, Saier Biotechnology, Tianjin) for 20 min in the dark. Then, harvested cells were resuspended in serum‐free cell culture medium. The fluorescence was determined by FACS cytometry (BD FACSAria II; BD Biosciences, San Jose, CA, USA).

### 2.4 Immunofluorescence

U251 and U251TR cells were seeded on glass coverslips in 24‐well plates and cultured for 24 h. After washing with PBS, cells were fixed with 4% paraformaldehyde for 30 min, permeabilized with 0.5% Triton X‐100 on ice for 5 min, and blocked with 10% donkey serum (in PBS) for 30 min at room temperature. Primary antibodies (1:50, diluted in 1% donkey serum) were incubated overnight at 4°C, followed by FITC‐conjugated secondary antibodies (1:200) for 2 h in the dark. After washing with PBS, nuclei were stained with DAPI (1:1000) for 5 min, and images were captured using the fluorescence microscope (Olympus).

### 2.5 Transmission Electron Microscopy (TEM)

U251/U251TR cells (3 × 10^6^ cells/T25 flask) were drug‐treated for 24 h, then harvested by trypsinization, centrifuged (1000 rpm, 10 min), and washed with PBS. Cells were fixed with 2.5% glutaraldehyde (2 h) and 1% osmium tetroxide (3 h), with 3 × 15‐min PBS washes after each step. Dehydration at 4°C used ethanol/acetone gradients (50%, 70%, 90% ethanol; 90% ethanol: acetone [1:1]; 90% acetone, 20 min each) followed by 100% acetone (3 × 20 min, RT). Embedding involved acetone: epoxy resin (2:1 for 4 h, 1:1 overnight, pure resin at 37°C for 3 h) and polymerization (37°C overnight, 45°C for 12 h, 60°C for 24 h). 50‐nm sections were stained with 1%−3% uranyl acetate (20−30 min) and lead citrate (pH 12, CO_2_‐free), then imaged by TEM.

### 2.6 CCK‐8 Assay

The cell proliferation assay was conducted using the CCK‐8 method. Cells were inoculated in a 96‐well microdroplet plate at a density of 2000 cells per well. The next day, the cells were treated with a gradient dilution of HOE‐642. Cell viability was detected by CCK8 at 24 and 48 h, respectively. Subsequently, 10 μL of CCK‐8 solution was added to each well, and the culture continued for 1 h. The absorbance was measured at 450 nm using Bio‐Tek's microplate reader.

### 2.7 pHi Measurements

The cell suspensions were prepared with HEPES at the cell concentration of 4 × 10^7^ cells/ml. We added BCECF‐AM/DMSO solution to the suspension to a final concentration of 3 µM. The fluorescence values of OD1 (440 nm) and OD2 (490 nm) were detected by a multifunctional microplate reader. The fluorescence ratio (FIR = OD2/OD1) reflects the pH and thus the activity of NHE1.

### 2.8 Animal Experiment

Nude mice were obtained from Saier Biotechnology. The 5 × 10^6^/ml U251TR cells suspended in 100 µL medium were injected subcutaneously on the left back of nude mice. Two weeks later, local multi‐point injections of TMZ + PBS and TMZ + HOE‐642 were given once every 3 days. The weight of the nude mice and the volume of the transplanted tumor were measured every 3 days. The nude mice were killed 2 days after drug withdrawal; the transplanted tumor was weighed, and the volume of the transplanted tumor was measured with a vernier caliper (V = 1/2 long diameter × short diameter^2^). After 3‐5 weeks of observation, according to the difference of tumor formation between the two groups, the nude mice were anesthetized and photographed; The tumor was completely removed, weighed, and photographed (scale). The tumor specimens were divided into two parts, one fresh tumor tissue was preserved at −80°C, and the other was fixed with neutral formalin. All studies performed with mice were approved by the Animal Care Committee of North China University of Science and Technology. All experiments involving mice complied with local and international regulations and ethical guidelines.

### 2.9 Immunohistochemistry (IHC)

5 μm paraffin sections were deparaffinized and rehydrated, followed by antigen retrieval in preheated citrate buffer (pH 6.0) at 95°C for 10–15 min. Endogenous peroxidase activity was blocked with 3% H_2_O_2_ at room temperature. Non‐specific binding was blocked with normal goat serum for 20 min. Sections were incubated with diluted primary antibody (1:100) overnight at 4°C, then washed with PBS and incubated with HRP‐conjugated secondary antibody for 20 min. DAB substrate was applied for chromogenic reaction under microscopic monitoring. After hematoxylin counterstaining and hydrochloric acid differentiation, slides were dehydrated, cleared in xylene, and sealed with neutral balsam. Protein expression was visualized and photographed under a microscope.

### 2.10 Enzyme‐Linked Immunosorbent Assay (ELISA)

Serum ALT and AST levels were measured by ELISA following the manufacturer's instructions. Standards and serum samples were incubated at 37°C for 30 min. After washing, the enzyme conjugate was added for secondary incubation. Following further washing, chromogens A and B were added for 15 min color development in the dark. The reaction was terminated, and absorbance at 450 nm was recorded within 15 min. Concentrations were calculated from standard curves, with all samples assayed in triplicate.

### 2.11 Hematoxylin‐Eosin (H&E) Staining

Paraffin‐embedded tumor tissue sections (5 μm) were deparaffinized in xylene and rehydrated via graded ethanol series. Sections were stained with Harris hematoxylin for nuclear labeling, differentiated in 1% hydrochloric acid alcohol, and blued under running tap water. Cytoplasmic staining was performed with eosin working solution. After serial dehydration and xylene clearing, slides were mounted with neutral balsam and observed under an optical microscope for histological morphology evaluation.

### 2.12 Statistical Analysis

All statistical analyses were performed using SPSS software version 17.0. The data were presented as the mean ± standard deviation (SD). One‐way analysis of variance was adopted to compare differences among groups. Scheffé post hoc testing was used for pairwise comparisons. *p* < 0.05 was considered to indicate a statistically significant difference.

## 3 Results

### 3.1 TMZ‐Resistant U251TR Cells Displayed Elevated Autophagy Levels and Reduced Apoptotic Rates

**Figure 1:**
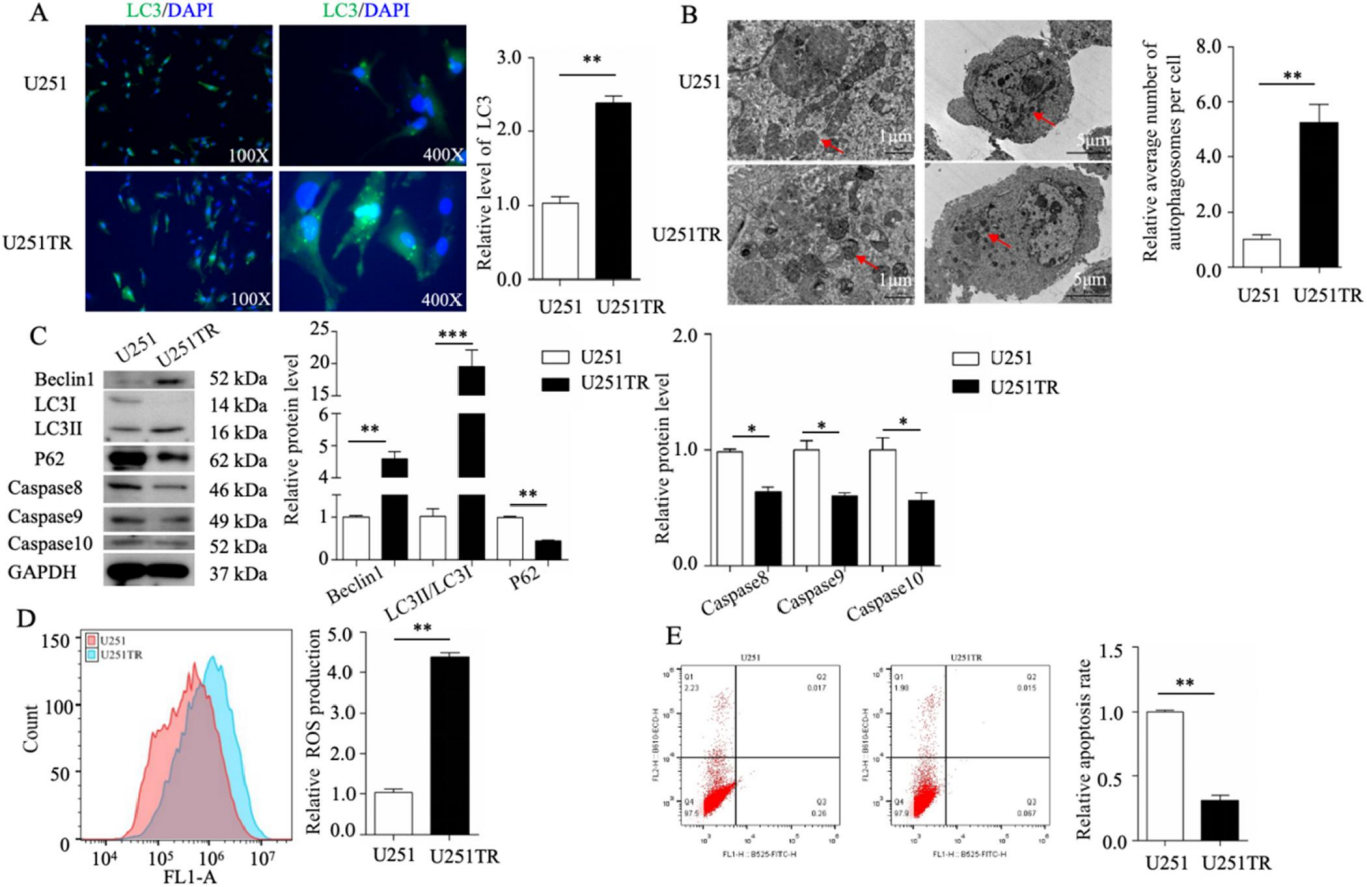
The levels of apoptosis and autophagy in U251 and TMZ‐resistant U251 (U251TR) cells. (A) Autophagosome formation of LC3 (Microtubule‐associated protein 1 light chain 3, a marker of autophagosomes) in the process of autophagy in U251 and U251TR cells was detected by immunofluorescence assay. (B) Autophagosomes in U251 and U251TR cells were observed by transmission electron microscopy. (C) The relative protein levels of core marker for autophagy initiation Beclin1, precursor marker for autophagosome formation LC3I, specific marker for autophagosome formation and maturation LC3II, marker for autophagic degradation efficiency P62, initiator of the extrinsic apoptotic pathway caspase 8, initiator of the intrinsic apoptotic pathway caspase 9, co‐initiator of exogenous apoptotic pathways caspase 10 in U251 and U251TR cells were analyzed by Western blotting. (D) The levels of reactive oxygen species (ROS) in U251 and U251TR cells were detected by flow cytometry. (E) The levels of apoptotic rate in U251 and U251TR cells was detected by flow cytometry. Results were shown as relative to the control. *p < 0.05, **p < 0.01.

Immunofluorescence staining for LC3, an autophagosome marker, revealed that the number of LC3‐positive puncta in U251TR cells was markedly higher than that in U251 cells (*p* < 0.01) (Figure 1A). TEM results demonstrated that there were increased autophagosomes (red arrows) in U251TR cells (Figure 1B). Compared with U251 cells, the expression of Beclin1 protein, a core initiator of autophagy, was significantly upregulated in U251TR cells (*p* < 0.01) (Figure 1C). Additionally, the LC3II/LC3I ratio was notably elevated (*p* < 0.01), which indicated enhanced conversion of LC3I to LC3II in U251TR cells (Figure 1C). Conversely, the protein level of P62, a substrate degraded during autophagy, was significantly lower in U251TR cells than in U251 cells (*p* < 0.01) (Figure 1C). These results suggested that autophagic activity was elevated in U251TR cells compared to U251 cells.

The results of flow cytometry showed that the ROS level in U251TR cells was markedly increased compared with that in U251 cells, indicating that U251TR cells are in a state of higher oxidative stress (*p* < 0.01) (Figure 1D). The apoptotic rate of U251TR cells was significantly lower than that of U251 cells (*p* < 0.01) (Figure 1E). The levels of caspase 9, a key protein in intrinsic apoptosis (a key mediator of intrinsic mitochondrial apoptosis), and caspase 8 and caspase 10, key proteins in extrinsic apoptosis (key mediators of extrinsic death receptor‐mediated apoptosis), were all downregulated in U251TR cells compared with those in U251 cells (*p* < 0.05, *p* < 0.05, *p* < 0.05) (Figure 1C). Western blot analysis revealed significantly elevated NHE1 and p‐NHE1 in U251TR cells compared with U251 cells (*p* < 0.05; *p* < 0.01) (Supporting Information S1: Figure 1), supporting subsequent NHE1 inhibition using HOE‐642. The above results indicated that U251TR cells have higher autophagy levels, higher ROS levels, and lower apoptotic rates than U251 cells.

### 3.2 NHE1 Inhibitor HOE‐642 Suppressed Cell Viability and ROS Levels, While Promoting Apoptosis in U251TR Cells

**Figure 2:**
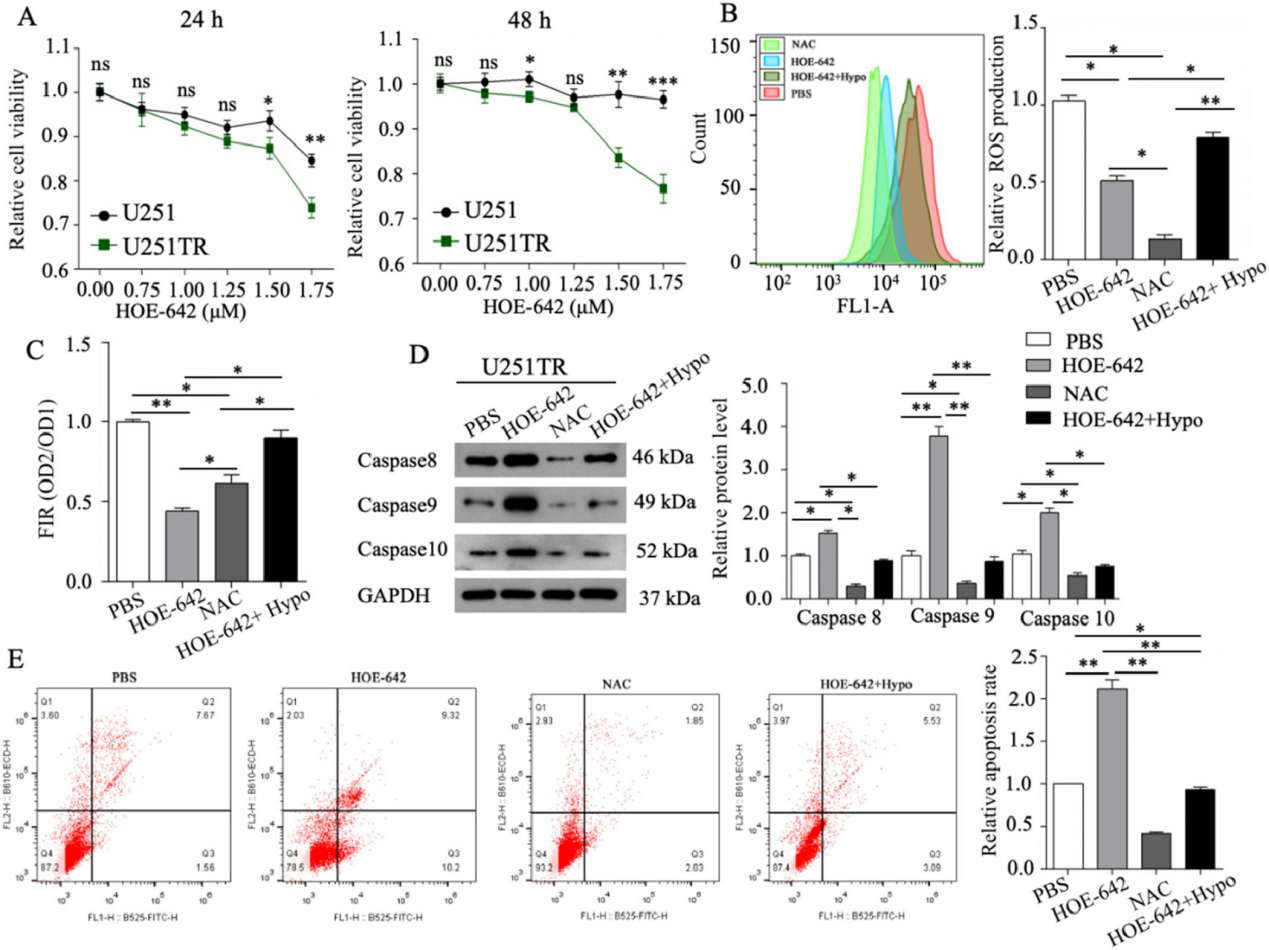
The effect of NHE1 inhibitor HOE‐642 on the viability, apoptosis and ROS levels in U251TR cells. (A) The cell viabilities of U251 and U251TR cells treated with HOE‐642 at different concentrations for 24 and 48 h was analyzed by CCK‐8. (B) The ROS levels of U251TR cells in each group were detected by flow cytometry. (C) The pH value, which reflects the NHE1 activity of U251TR cells in each group, was detected by the BCECF‐AM fluorescent probe. The Fluorescence Intensity Ratio (FIR, FIR = OD2/OD1) at 440 nm and 490 nm was measured by UV‐Visible, which reflects the pH value of each group. HOE‐642 is an NHE1 inhibitor, N‐Acetylcysteine (NAC) is an inhibitor of ROS, hypoxic treatment (Hypo) for 24 h is adopted as a physiological activator of ROS. (D) The relative protein levels of extrinsic apoptotic pathway caspase 8, initiator of the intrinsic apoptotic pathway caspase 9, co‐initiator of exogenous apoptotic pathways caspase 10 of U251TR cells in each group were analyzed by Western blotting. (E) The apoptotic rates of U251TR cells in each group were detected by flow cytometry. Results were shown as relative to control. *p < 0.05, **p < 0.01, ***p < 0.005.

The viability of U251 and U251TR cells treated with different concentrations of NHE1 inhibitor HOE‐642 for 24 h and 48 h was detected by the CCK‐8 assay. The result indicated that with increasing concentrations of HOE‐642, the viabilities of both U251 and U251TR cells showed a downward trend (Figure 2A). The optimal non‐cytotoxic concentration (1.00 μM) and treatment time (24 h) of HOE‐642 were selected for subsequent experiments (Figure 2A). Flow cytometry analysis showed that HOE‐642 significantly reduced ROS levels in U251TR cells (*p* < 0.05), although the effect was not as good as the ROS inhibitor NAC (*p* < 0.05) (Figure 2B). However, hypoxic treatment (Hypo) for 24 h(physiological activator of ROS) reversed the decrease in ROS level induced by HOE‐642 (*p* < 0.05). The BCECF‐AM fluorescent probe results showed that both HOE‐642 and NAC significantly inhibited NHE1 activity in U251TR cells (*p* < 0.01, *p* < 0.05). Hypoxic treatment markedly reversed the inhibition of NHE1 activity by HOE‐642 (*p* < 0.05) (Figure 2C). These results indicated a positive feedback loop between NHE1 activity and ROS levels in U251TR cells.

HOE‐642 significantly promoted apoptosis in U251TR cells (*p* < 0.01), while NAC exerted a strong inhibitory effect on apoptosis (*p* < 0.01) (Figure 2E). Importantly, Hypoxic treatment markedly attenuated cellular apoptosis rate induced by HOE‐642 (*p* < 0.01) (Figure 2E). The protein levels of apoptotic proteins caspase 8, caspase 9, and caspase 10 in U251TR cells were all significantly up‐regulated by HOE‐642 (*p* < 0.05, *p* < 0.01, *p* < 0.05) (Figure 2D). In contrast, the ROS inhibitor NAC exerted the opposite effect (*p* < 0.05, *p* < 0.05, *p* < 0.05) (Figure 2D). Notably, hypoxic treatment significantly reversed the up‐regulation of the three caspase proteins caused by HOE‐642 (*p* < 0.05, *p* < 0.01, *p* < 0.05) (Figure 2D). This might be attributed to hypoxia‐mediated partial restoration of ROS levels (as shown in Figure 2B), which in turn modulates apoptotic signaling or other NHE1‐related pathways to counteract HOE‐642‐induced apoptosis. In summary, the NHE1 inhibitor HOE‐642 could reduce the activity of NHE1, suppress the level of ROS, as well as promote apoptosis of GBM cells, through the NHE1‐ROS positive feedback mechanism.

### 3.3 NHE1 Inhibitor HOE‐642 Blocked Autophagy in U251TR Cells

**Figure 3:**
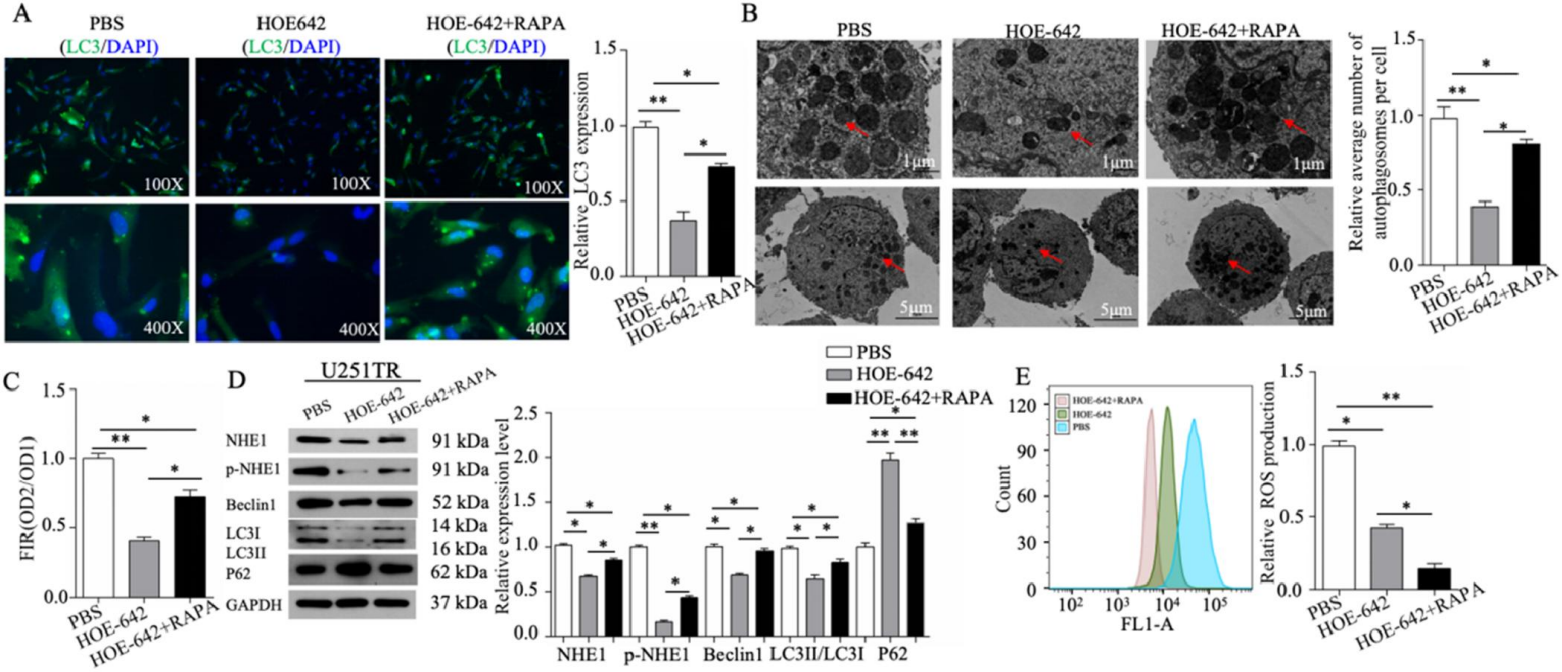
The effects of NHE1 inhibitor HOE‐642 on autophagy in U251TR cells. The U251TR cells were treated with PBS, HOE‐642, HOE‐642 + RAPA (Rapamycin, an autophagy inducer), respectively. (A) The LC3 autophagosome formation of U251TR cells in each group was detected by immunofluorescence assay. (B) The autophagosomes of U251TR cells in each group were observed by transmission electron microscopy. The U251TR cells were treated with PBS, HOE‐642, NAC, and NAC + RAPA, respectively. (C) The NHE1 activity of U251TR cells in each group was detected by BCECF‐AM fluorescent probe. FIR indicates the pH value, which indirectly reflects the activity of NHE1 in the cell. (D) The relative protein levels of NHE1, p‐NHE1, and core marker for autophagy initiation Beclin1, precursor marker for autophagosome formation LC3I, specific marker for autophagosome formation and maturation LC3II, marker for autophagic degradation efficiency P62 of U251TR cells in each group were analyzed by Western blotting. Results were shown as relative to control. *p < 0.05, **p < 0.01.

The immunofluorescence staining results revealed that LC3‐positive puncta were significantly decreased in the HOE‐642 group (*p* < 0.01), while LC3‐positive puncta were markedly increased in the HOE‐642 + RAPA (autophagy inducer) group (*p* < 0.05) (Figure 3A). Similarly, as shown in Figure 3B, TEM showed that the number of autophagosomes was reduced in U251TR cells after HOE‐642 treatment, while the number of autophagosomes was increased in the HOE‐642 + RAPA group (*p* < 0.05). These results indicated that HOE‐642 could inhibit autophagy in U251TR cells.

NHE1 activity was significantly reduced in the HOE‐642 group (*p* < 0.01), while it was partially recovered by the autophagy inducer RAPA (*p* < 0.01) (Figure 3C). Compared with the PBS group, both the NHE1 protein and p‐NHE1 levels were markedly down‐regulated in the HOE‐642 group (*p* < 0.05, *p* < 0.01) (Figure 3D). The Beclin1 level, a key driver of autophagy initiation, was significantly decreased by HOE‐642 treatment (*p* < 0.05) (Figure 3D). The LC3II/LC3I ratio, reflecting autophagosome formation, was decreased (*p* < 0.05) (Figure 3D). The autophagic substrate P62 was significantly accumulated in the HOE‐642 group compared with that in the PBS group (*p* < 0.01) (Figure 3D). Following co‐treatment with HOE‐642 and RAPA, the expression patterns of these proteins were partially reversed (NHE1, *p* < 0.05; p‐NHE1, *p* < 0.05; Beclin1, *p* < 0.05; P62, *p* < 0.01) and the ratio of LC3II/LC3I increased (*p* < 0.05). To further verify the changes in autophagic flux, chloroquine (CQ) was used to block lysosomal degradation (Supporting Information S1: Figure 2). CQ‐induced LC3II accumulation was markedly attenuated following HOE‐642 intervention (*p* < 0.05), confirming that HOE‐642 suppresses autophagosome formation at the initiation stage rather than impairing lysosomal clearance. These data suggested that HOE‐642 inhibited the initiation and flux of autophagy. There may be a functional interaction between NHE1 phosphorylation and autophagy pathways, and RAPA could counteract the inhibitory effect of HOE‐642 on autophagy. The autophagy inducer RAPA potentiated the suppressive effect of HOE‐642 against ROS production (*p* < 0.05) (Figure 3E). These findings provided a basis for exploring the regulatory mechanism of the NHE1‐ROS‐autophagy signaling axis.

### 3.4 NHE1 Regulated Autophagy by Influencing ROS and Downstream Signaling Pathways

**Figure 4:**
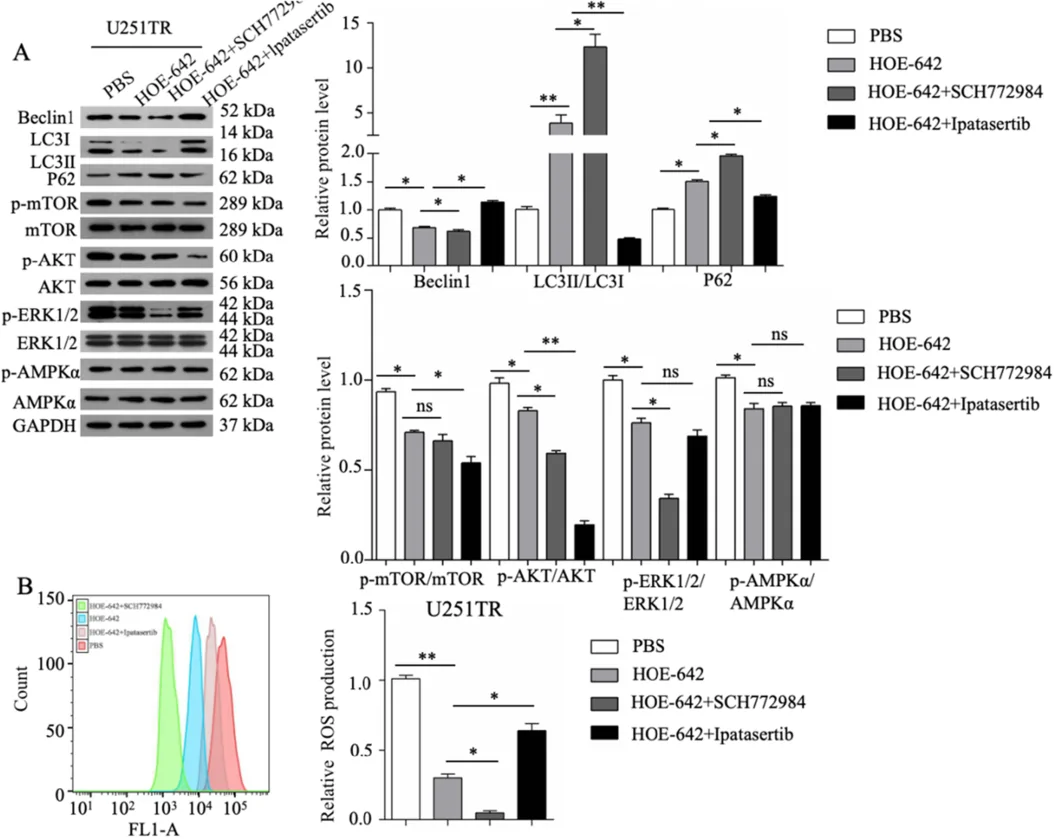
The effects of NHE1 inhibitor HOE‐642 on ROS and downstream signaling pathways in U251TR cells. The U251TR cells were treated with PBS, HOE‐642, HOE‐642 + SCH772984 (ERK inhibitor), HOE‐642 + Ipatasertib (AKT inhibitor), respectively. (A) The relative protein levels of core marker for autophagy initiation Beclin1, precursor marker for autophagosome formation LC3I, specific marker for autophagosome formation and maturation LC3II, marker for autophagic degradation efficiency P62, p‐mTOR, mTOR, p‐Akt, Akt, p‐ERK1/2, ERK1/2, p‐AMPKα, AMPKα of U251TR cells in each group were analyzed by Western blotting. (B) The ROS levels of U251TR cells in each group were detected by flow cytometry. SCH772984 is a novel specific ERK inhibitor, Ipatasertib is an AKT inhibitor. Results were shown as relative to control. *p < 0.05, **p < 0.01.

The protein level of Beclin1 in the HOE‐642 group was significantly lower than that in the PBS group (*p* < 0.05). Correspondingly, the LC3II/LC3I ratio was also reduced (*p* < 0.01), whereas the P62 protein level was markedly elevated (*p* < 0.05) (Figure 4A). ERK inhibitor SCH772984 markedly enhanced the inhibitory effect of HOE‐642 on Beclin1 (*p* < 0.05). The LC3II/LC3I ratio was also reduced (*p* < 0.05), with a corresponding significant elevation in the P62 protein level (*p* < 0.05). However, the AKT inhibitor Ipatasertib markedly reversed the inhibitory effect of HOE‐642 on Beclin1 (*p* < 0.01); the LC3II/LC3I ratio also increased (*p* < 0.01), alongside a marked decline in P62 protein level (*p* < 0.05). These results indicated that HOE‐642 reduced autophagy activity by down‐regulating the expression of autophagy‐related proteins and inhibiting autophagic flux. The ERK and AKT signaling pathways mediated the effects of HOE‐642 on autophagy, with ERK promoting and AKT suppressing autophagy.

The p‐mTOR/mTOR ratio showed no significant difference between the HOE‐642 and HOE‐642 + SCH772984 groups (*p* > 0.05) (Figure 4A), but both were significantly lower than the PBS group (*p* < 0.05). This ratio was further reduced in the HOE‐642+Ipatasertib group (*p* < 0.05) (Figure 4A). It indicated that HOE‐642 significantly inhibited mTOR pathway activity. The ERK inhibitor SCH772984 had no effect on mTOR pathway. The AKT inhibitor Ipatasertib could further enhance the inhibitory effect of HOE‐642 on the mTOR pathway. Although HOE‐642 inhibited mTOR activity, it eventually inhibited autophagy, indicating that the down‐regulation of mTOR pathway is not the core mechanism of autophagy regulation by HOE‐642, and the inhibitory effect on autophagy might be dominated by AKT pathway.

The ratio of p‐AKT/AKT in the HOE‐642 group was significantly lower than that in the PBS group (*p* < 0.05). In the HOE‐642 + SCH772984 group, this proportion decreased more significantly (*p* < 0.05). The HOE‐642+Ipatasertib group further significantly reduced this proportion (*p* < 0.01). It indicated that HOE‐642 significantly inhibited AKT pathway activity. The ERK inhibitor SCH772984 could synergistically enhance the inhibitory effect of HOE‐642 on the AKT pathway, suggesting a positive cross‐regulatory relationship between the ERK and AKT pathways in the HOE‐642‐mediated regulatory network. The more significant reduction in the p‐AKT/AKT ratio in the HOE‐642+Ipatasertib group indicated that Ipatasertib exerted a stronger specific inhibitory effect on AKT activity.

The p‐ERK1/2/ERK1/2 ratio in the HOE‐642 group was significantly lower than that in the PBS group (*p* < 0.05). The HOE‐642 + SCH772984 group was further significantly reduced (*p* < 0.05). Compared with the HOE‐642 group, there was no significant change in the above proportions in the HOE‐642+Ipatasertib group (*p* > 0.05). It indicated that HOE‐642 significantly inhibited the activity of ERK1/2 pathway, which was synergistically enhanced by ERK inhibitor SCH772984, while AKT inhibitor Ipatasertib had no effect on HOE‐642‐mediated ERK1/2 pathway inhibition. This result further supported that the inhibition of the ERK1/2 pathway was a core mechanism for HOE‐642 to suppress autophagy, and this regulation was not affected by the intervention of the AKT pathway.

The ratio of p‐AMPK α/AMPKα in the HOE‐642 group was significantly lower than that in the PBS group (*p* < 0.05). There was no significant difference between the HOE‐642 + SCH772984 or HOE‐642+Ipatasertib and HOE‐642 groups (*p* > 0.05, *p* > 0.05). It indicated that HOE‐642 significantly inhibited the activity of AMPKα pathway, but this effect was not affected by ERK inhibitor SCH772984 and AKT inhibitor Ipatasertib. AMPKα pathway was not involved in the regulation of autophagy mediated by HOE‐642.

When HOE‐642 was combined with SCH772984, the relative ROS production was lower than that of the HOE‐642 group and significantly lower than that of the PBS group (*p* < 0.05, *p* < 0.05) (Figure 4B). However, in the HOE‐642+Ipatasertib group, the relative ROS production was significantly higher than that in the HOE‐642 group and the HOE‐642 + SCH772984 group (*p* < 0.05, *p* < 0.05), but still lower than that in the PBS group (*p* < 0.05). The results showed HOE‐642 significantly inhibited intracellular ROS production, which was synergistically enhanced by ERK inhibitor SCH772984 and alleviated by AKT inhibitor Ipatasertib. The changes in ROS production were completely synchronized with the previous changes in AKT/ERK pathway regulation and autophagy activity, suggesting that ROS might be a key upstream mediator of AKT/ERK pathway mediating HOE‐642 regulation of autophagy.

To further clarify the distinct roles of AKT and ERK in autophagy regulation, cells were treated with ipatasertib or SCH772984 alone (Supporting Information S1: Figure 3). Ipatasertib inhibited AKT phosphorylation and promoted autophagy, while SCH772984 suppressed ERK activation and attenuated autophagy. These data further confirmed the antagonistic effects of AKT and ERK on autophagy.

### 3.5 HOE‐642 Enhanced the Sensitivity of GBM to TMZ In Vivo

**Figure 5:**
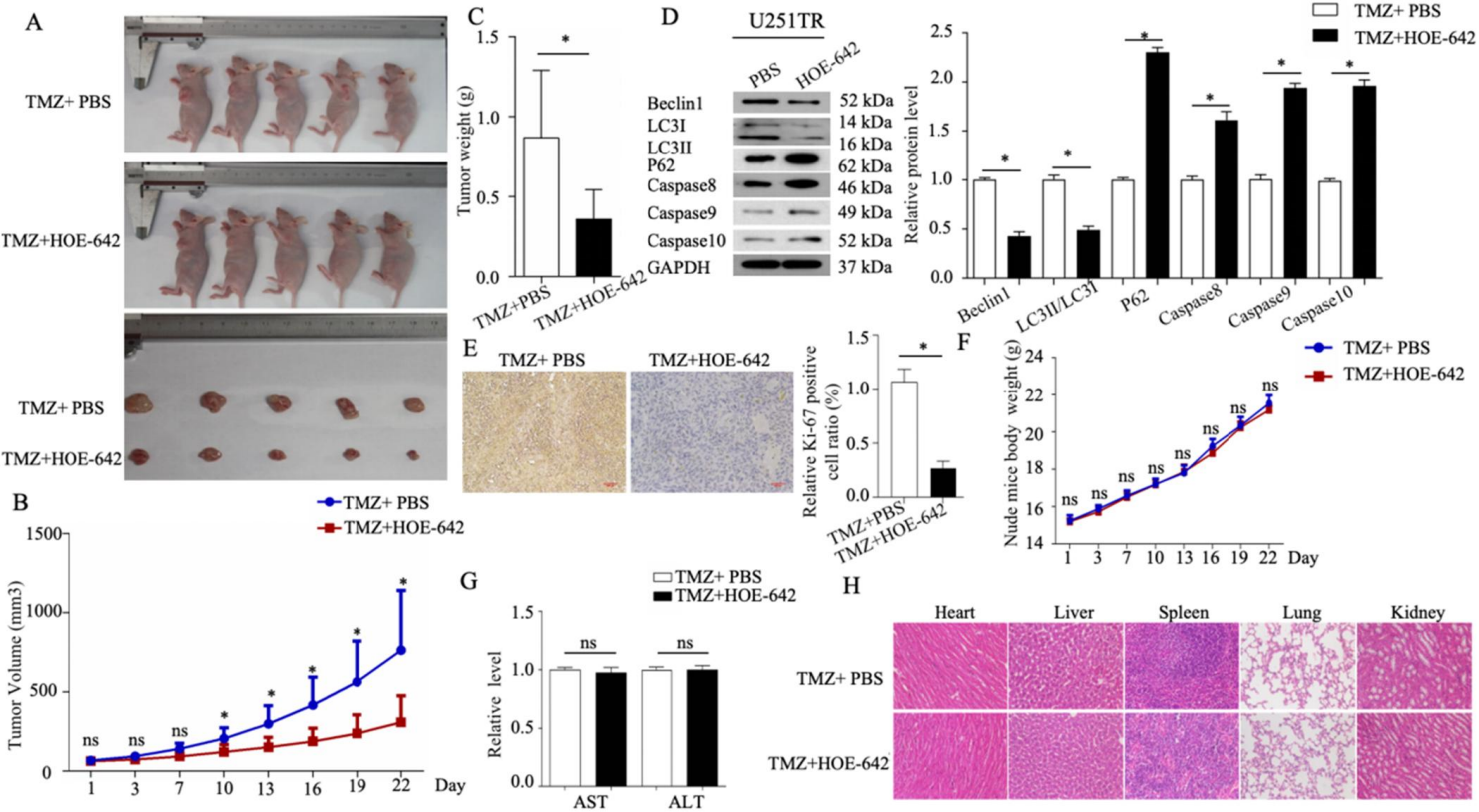
The effect of NHE1 inhibitor HOE‐642 on TMZ sensitivity of GBM cells was analyzed in vivo. The nude mice were treated with TMZ + PBS, TMZ + HOE‐642, respectively. (A) In vivo tumor formation assay. Representative whole‐body photographs of nude mice and excised tumor images in each group. (B) The nude mouse body tumor growth curve. The long diameter and short diameter of the tumor were measured and weighed every 3 days, and the growth curve of the transplanted tumor was drawn based on the average tumor volume of each group of animals. (C) Statistics of tumor weight in each group of nude mice. (D) Representative IHC staining of Ki‐67 in tumor tissues and quantitative statistics of Ki‐67 positive cell ratio. (E) The relative protein levels of core marker for autophagy initiation Beclin1, precursor marker for autophagosome formation LC3I, specific marker for autophagosome formation and maturation LC3II, marker for autophagic degradation efficiency P62 and extrinsic apoptotic pathway caspase 8, initiator of the intrinsic apoptotic pathway caspase 9, co‐initiator of exogenous apoptotic pathways caspase 10 in tumor tissues of each group were analyzed by Western blotting. (F) Dynamic body weight monitoring of nude mice. (G) Serum AST and ALT levels were detected by ELISA. (H) Representative H&E staining of major organs (heart, liver, spleen, lung, kidney). Results were shown as relative to control. *p < 0.05, **p < 0.01.

Compared with the TMZ + PBS group, the TMZ + HOE‐642 group exhibited significantly reduced tumor volume (Figure 5A,B), accompanied by a slower tumor growth rate (Figure 5B), and decreased tumor weight (*p* < 0.05) (Figure 5C). The relative ratio of Ki‐67‐positive proliferative cells was significantly decreased in the TMZ + HOE‐642 group (*p* < 0.05) (Figure 5D). The protein level of the autophagy‐promoting marker Beclin1 and the LC3II/LC3I ratio were significantly decreased (*p* < 0.05; *p* < 0.05). Meanwhile, the protein level of the autophagic substrate P62 was significantly increased (*p* < 0.05) (Figure 5E), indicating weakened autophagy flux in the TMZ + HOE‐642 group. The apoptotic proteins caspase 8, caspase 9 and caspase 10 were significantly upregulated in the TMZ + HOE‐642 group (*p* < 0.05, *p* < 0.05, *p* < 0.05) (Figure 5E). The body weight of nude mice exhibited no significant difference between the two groups at all time points (all *p* > 0.05) (Figure 5F). ELISA detection showed no significant differences in serum AST and ALT levels between the two groups (all *p* > 0.05) (Figure 5G), suggesting that the combined treatment exerted no obvious hepatic toxicity. Consistently, histological analysis via H&E staining further demonstrated no apparent pathological lesions or tissue damage in the major vital organs, including the heart, liver, spleen, lung, and kidney (Figure 5H). Collectively, these results indicated that the combination of TMZ and HOE‐642 exhibited good in vivo biosafety with no obvious systemic toxicity. These data suggested that HOE‐642 enhanced GBM sensitivity to TMZ in vivo by inhibiting autophagy and activating apoptosis, thereby inhibiting tumor growth, without inducing evident organ injury or systemic toxicity.

### 3.6 Diagram Showing the Mechanism of NHE1‐induced Autophagy in GBM

**Figure 6:**
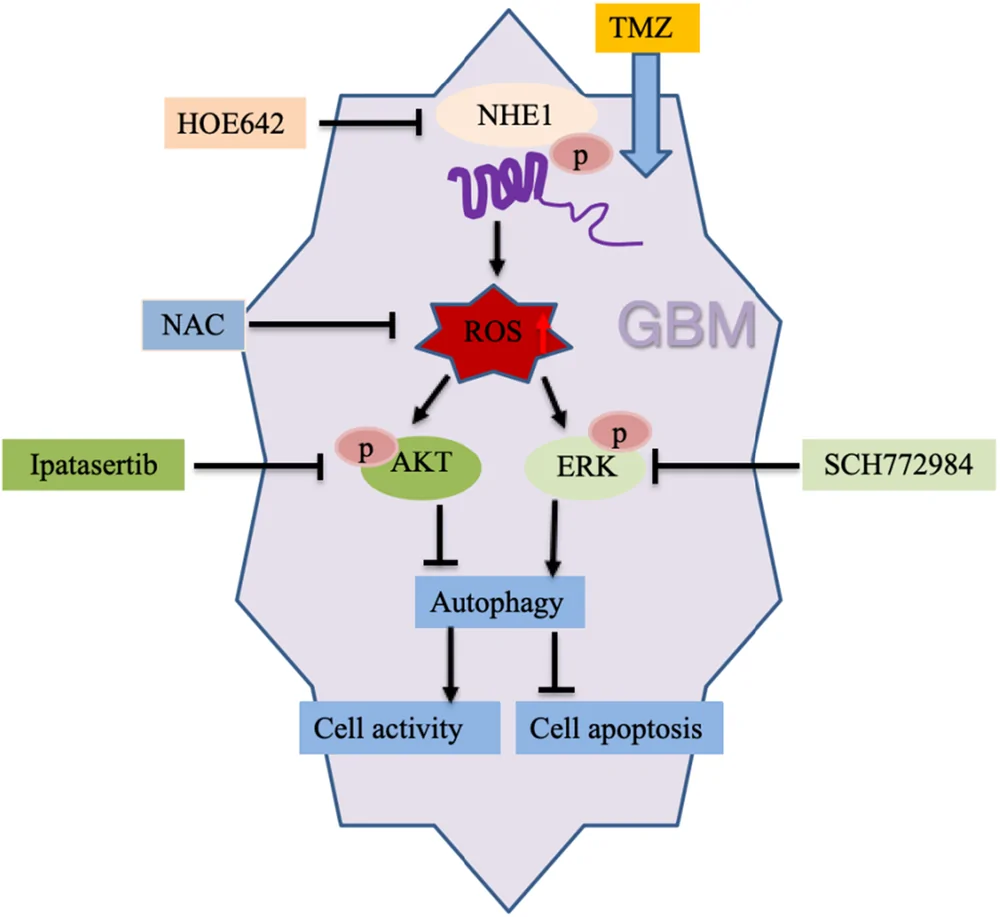
Schematic diagram illustrating the NHE1‐ROS‐AKT/ERK‐autophagy regulatory axis underlying HOE‐642‐mediated TMZ sensitization in TMZ‐resistant GBM cells. Overactivated NHE1 increases intracellular ROS accumulation, which triggers persistent phosphorylation of AKT and ERK. Phosphorylated ERK facilitates autophagic activity, whereas phosphorylated AKT suppresses autophagy. The balanced crosstalk between AKT and ERK promotes continuous protective autophagy, which enables tumor cells to evade TMZ‐induced apoptosis and ultimately develop chemoresistance. Treatment with the NHE1 inhibitor HOE‐642 reduces ROS generation, decreases AKT and ERK phosphorylation, disrupts autophagic flux and restores TMZ‐initiated apoptotic responses. Additional intervention reagents (NAC, SCH772984, Ipatasertib) target distinct nodes of this axis to modulate autophagy and cell fate in drug‐resistant GBM cells. NAC, N‑Acetylcysteine; NHE1, Na+/H+ exchanger 1; ROS, reactive oxygen species; TMZ, Temozolomide.

To elucidate the effect and molecular mechanism of the microenvironmental regulator NHE1 on autophagy in drug‐resistant GBM cells, we constructed a signaling network centered on the NHE1‐ROS axis, as shown in Figure 6. Mechanistically, activated NHE1 (p‐NHE1), promotes intracellular ROS production. The accumulated ROS further activates the p‐AKT and p‐ERK signaling cascades. p‐ERK promotes autophagy by upregulating autophagy‐related proteins including Beclin1 and LC3I/II and downregulating P62, while p‐AKT exerts the opposite effect. Together, these pathways modulate autophagy activity, and changes in this process in turn regulate the viability and apoptosis of drug‐resistant GBM cells.

Several agents target discrete nodes within this NHE1‐ROS‐AKT/ERK‐autophagy axis: HOE‐642 directly inhibits NHE1 and p‐NHE1, reducing intracellular ROS, downregulating p‐AKT and p‐ERK, and suppressing autophagy; TMZ modulates the activation status of NHE1 in resistant GBM cell. NAC scavenges intracellular ROS, thereby blocking downstream activation of p‐AKT and p‐ERK. SCH772984, an ERK inhibitor, specifically targets p‐ERK, synergistically enhancing HOE‐642's autophagy‐suppressive effects, while also further lowering p‐AKT levels, indicating positive cross‐regulation between the ERK and AKT pathways. Ipatasertib, an AKT inhibitor, targets p‐AKT, reversing HOE‐642‐induced autophagy suppression while attenuating HOE‐642's ability to reduce intracellular ROS.

In summary, this schematic outlines the complete regulatory network in drug‐resistant GBM: the NHE1‐ROS‐AKT/ERK axis acts as the core pathway mediating autophagy regulation, and targeted intervention at distinct nodes of this axis using the agents described modulates the expression of key autophagy‐related proteins to govern the fate of drug‐resistant GBM cells.

## 4 Discussion

Focusing on NHE1, this study systematically analyzed its regulatory effects on autophagy, ROS homeostasis, and apoptosis in TMZ‐resistant GBM cells. We first identified the novel NHE1‐ROS‐AKT/ERK‐autophagy regulatory axis, clarified its molecular mechanism in the modulation of TMZ sensitivity, provided new theoretical insights for addressing GBM chemoresistance, and confirmed NHE1 as a potential therapeutic target‐both in vitro and in vivo.

Enhanced autophagic flux confers chemoresistance across tumors, and targeting the lysosome‐autophagy cascade represents a universal strategy to reverse drug tolerance [33]. In gastrointestinal cancers, autophagy‐driven drug insensitivity has been well documented: quercetin restrains excessive autophagy to restore 5‐FU sensitivity in colorectal cells [34], whereas lncRNA WT1‐AS remodels PI3K/Akt/mTOR signaling to promote chemoresistance in gastric cancer [35]. Beyond digestive malignancies, comparable resistance mechanisms exist in other epithelial tumors: botanical extracts block protective autophagy to reverse chemotherapy failure in colorectal models [36], and ARRDC3 overexpression sustains autophagic flux to induce cisplatin resistance in lung cancer [37]. Inspired by these pan‐cancer findings, we hypothesized that analogous pro‐survival autophagy enables GBM cells to evade TMZ cytotoxicity. To verify this, we comprehensively profiled disparities in autophagy activity, oxidative stress, and apoptotic cascades between U251 and U251TR cells. Our results showed that U251TR cells exhibited significantly elevated autophagic activity compared with U251 cells. Immunofluorescence staining revealed a marked increase in LC3‐positive puncta, and TEM demonstrated significant autophagosome accumulation. Molecularly, Beclin1 (a key autophagy‐initiating protein) was upregulated, LC3I to LC3II conversion was enhanced, and the autophagic substrate P62 was downregulated. Meanwhile, the apoptosis rate of U251TR cells was significantly reduced, and the expressions of caspase 9 (a key protein in the intrinsic apoptotic pathway) and caspase 8/10 (key proteins in the extrinsic apoptotic pathway) were significantly downregulated. These findings indicate that U251TR cells evade TMZ‐induced cell death by activating protective autophagy, which is consistent with previous studies showing that autophagy activation mediates GBM resistance by maintaining cellular energy balance and reducing oxidative damage [38]. Notably, U251TR cells also showed significantly increased ROS levels, which aligns with previous studies demonstrating the involvement of oxidative stress in autophagy activation and GBM chemotherapy resistance [39, 40, 41, 42], suggesting close crosstalk between redox homeostasis and autophagic signaling in TMZ‐resistant GBM.

Recent GBM studies further elaborate the tight linkage between oxidative stress and drug‐resistant autophagy. Boric acid eliminates TMZ resistance via inhibiting NCOA4‐mediated ferritinophagy, while cromolyn impairs microglial ferritin through the identical NCOA4‐dependent pathway, revealing extensive crosstalk between iron metabolism, ROS, and autophagy in GBM [40, 41]. Moreover, Notch1 and major vault protein (MVP) jointly drive TMZ resistance independent of autophagy, providing an alternative resistance cascade parallel to our NHE1‐ROS‐autophagy axis [42]. Mitochondrial ROS homeostasis is tightly regulated by KATP channels; MitoQ disrupts mitochondrial integrity and suppresses cytoprotective autophagy by modulating KATP activity, strongly supporting our conclusion that ROS acts as an indispensable upstream signal governing autophagic activity [43].

NHE1 serves as a core molecule maintaining pHi homeostasis. It is frequently overexpressed or hyperactivated in tumor cells, leading to extracellular microenvironment acidification and intracellular alkalization, which in turn promote tumor progression and chemoresistance [44]. In the present study, NHE1 acts as a key regulator of the autophagy‐apoptosis balance in TMZ‐resistant GBM cells. Treatment with the NHE1 inhibitor HOE‐642 reduced the viability of U251TR cells, inhibited ROS production, and significantly increased apoptosis. More importantly, we identified a positive feedback loop between NHE1 activity and ROS levels: hypoxia, a physiological ROS activator, reversed both HOE‐642‐induced ROS reduction and NHE1 inhibition. This feedback loop likely sustains the survival of TMZ‐resistant GBM cells by preserving redox balance and pHi homeostasis. These observations align with prior studies showing that NHE1 and ROS interact to drive tumor progression [43, 45, 46]. HOE‐642 upregulated the expression of caspase 8, 9 and 10 and induced apoptosis, which further confirms that NHE1 acts as a key survival factor for TMZ‐resistant GBM cells. This finding indicates that NHE1 inhibition enhances the chemosensitivity of the glioma model.

The regulatory mechanism of NHE1 in autophagy is intricate, involving crosstalk between ROS and multiple signaling pathways [47]. HOE‐642 potently suppressed autophagic activity in U251TR cells, an effect that was partially reversed by the autophagy inducer RAPA, which also restored p‐NHE1 levels, suggesting a functional correlation between NHE1 activity and autophagic signaling cascades. Further mechanistic investigations revealed that NHE1 modulates autophagy primarily via the ROS‐AKT/ERK signaling axis. HOE‐642 downregulated the phosphorylation of AKT and ERK1/2 by reducing intracellular ROS levels. Specifically, the ERK pathway exerted a pro‐autophagic effect, and treatment with the ERK inhibitor SCH772984 synergistically enhanced the inhibitory effect of HOE‐642 on autophagy‐related proteins including Beclin1 and LC3II, while further reducing ROS production. In contrast, the AKT pathway exhibited an anti‐autophagic effect. Treatment with the AKT inhibitor Ipatasertib reversed HOE‐642‐induced autophagy inhibition and partially restored intracellular ROS levels. This antagonism between the ERK and AKT pathways forms a delicate balance that modulates autophagic flux in TMZ‐resistant GBM cells, consistent with previous reports that AKT and ERK exert opposing autophagy‐regulating functions across multiple tumor types [48, 49].

Although HOE‐642 also inhibited the mTOR and AMPKα pathways, these two pathways are not the core mediators of NHE1‐regulated autophagy. As a classical negative regulator of autophagy [50, 51], mTOR inhibition by HOE‐642 did not induce autophagy, indicating the AKT/ERK axis dominates autophagy regulation in this study. HOE‐642‐mediated inhibition of the AMPKα pathway remained unaffected by SCH772984 and Ipatasertib, suggesting it is not involved in NHE1‐mediated autophagic regulation. This finding clarifies the specificity of ROS‐AKT/ERK axis‐mediated autophagy suppression in TMZ‐resistant GBM, providing a precise direction for subsequent targeted therapy.

The results of in vivo experiments further verified the therapeutic potential of targeting NHE1. Compared with TMZ monotherapy, the combination of HOE‐642 and TMZ significantly reduced tumor volume, slowed tumor growth rate, and decreased tumor weight. Concurrently, in tumor tissues from the combination treatment group, Beclin1 expression levels was downregulated and P62 accumulated, indicating attenuated autophagic flux [52]. Meanwhile, the expressions levels of caspase 8, 9, and 10 were markedly upregulated, which reflected activated apoptosis [53]. These findings align with in vitro results, confirming that HOE‐642 enhances GBM sensitivity to TMZ by disrupting the autophagy‐apoptosis balance. Collectively, these data provide reliable preclinical evidence supporting NHE1 targeting as a viable strategy to overcome TMZ resistance in GBM patients.

This study identifies a novel NHE1‐ROS‐AKT/ERK‐autophagy regulatory axis in TMZ‐resistant GBM and clarifies the antagonistic roles of AKT and ERK in NHE1‐mediated autophagy regulation. In contrast to prior reports of NHE1 regulating autophagy via PI3K/Akt/mTOR in other cancers [23, 54], we demonstrate for the first time that NHE1 modulates autophagy through ROS‐dependent AKT/ERK crosstalk in TMZ‐resistant GBM, which offers a new mechanistic explanation for sustained high autophagic activity in drug‐resistant cells. Given that are based on the U251TR cell line, future work will validate this axis in additional TMZ‐resistant GBM cell lines and PDX models. Further investigations will focus on the synergistic effects of NHE1 inhibitors combined with ERK modulators to enhance TMZ sensitivity in GBM [55, 56]. In addition, combinatorial regimens pairing NHE1 inhibitors with ferroptosis inducers such as boric acid or cromolyn deserve deeper exploration, as simultaneous inhibition of autophagy and iron‐dependent survival signaling may produce superior anti‐tumor efficacy against TMZ‐resistant GBM [40, 41].

In conclusion, this study confirms that NHE1 acts as a key regulator of autophagy and apoptosis in TMZ‐resistant GBM cells by modulating ROS and the AKT/ERK signaling pathways. Targeting NHE1 with HOE‐642 disrupts the autophagy‐apoptosis balance, inhibits tumor growth, and enhances GBM cell sensitivity to TMZ both in vitro and in vivo. These findings deepen our understanding of the molecular mechanisms underlying TMZ resistance in GBM, providing new targets and strategies for improving therapeutic outcomes of this malignant disease.

## Author Contributions

**Dan Li:** conceptualization, methodology, data curation, investigation, formal analysis, writing – original draft, funding acquisition, visualization, writing – review and editing. **Yuhui Li:** conceptualization, investigation, funding acquisition, writing – original draft, methodology, visualization, formal analysis, data curation, writing – review and editing. **Xuan Zheng:** methodology, validation, writing – review and editing, software. **Zhuo Wang:** methodology, validation, writing – review and editing, software. **Yang Wang:** investigation, data curation, writing – review and editing. **Jun Zhang:** methodology, formal analysis, writing – review and editing. **Jianxiong Guo:** investigation, data curation, writing – review and editing. **Lei Wang:** investigation, methodology, writing – review and editing. **Jingwu Li:** formal analysis, validation, writing – review and editing. **Yongliang Liu:** writing – review and editing, conceptualization, supervision, project administration, resources. **Yufeng Li:** writing – review and editing, conceptualization, supervision, project administration, resources.

## Ethics Statement

All studies performed with mice were approved by the Animal Care Committee of North China University of Science and Technology. All experiments involving mice complied with local and international regulations and ethical guidelines.

## Conflicts of Interest

The authors declare no conflicts of interest.

## Supporting information


Supporting File

## Data Availability

The data that support the findings of this study are available from the corresponding author upon reasonable request.
