# Supplementary material for: Inhibition of NHE1 Overcomes Temozolomide‐Resistance in Glioblastoma via ROS‐AKT/ERK Axis‐Mediated Autophagy Suppression
Source: J Biochem Mol Toxicol. 2026 Sep 14;40(9):e71110. doi: 10.1002/jbt.71110 (PMC13575469; doi:10.1002/jbt.71110)
Supplement: Supplementary file 1 [file JBT-40-e71110-s001.docx]

**Supplementary Figure**

**Supplementary Figure S1. The levels of NHE1 and p-NHE1 in U251 and TMZ-resistant U251 (U251TR) cells**

The levels of NHE1, p-NHE1 in U251 and U251TR cells were analyzed by Western blotting. **P*<0.05, ***P*<0.01.

**Supplementary Figure S2. The levels of autophagy-related proteins in PBS/HOE-642/CQ-treated U251TR cells**

The U251TR cells were treated with PBS, HOE-642, CQ (chloroquine , classic lysosomal degradation inhibitor), HOE-642+CQ, respectively. The relative protein levels of core marker for autophagy initiation Beclin1, precursor marker for autophagosome formation LC3I, specific marker for autophagosome formation and maturation LC3II, marker for autophagic degradation efficiency P62 of U251TR cells in each group were analyzed by Western blotting. **P*<0.05, ***P*<0.01.

**Supplementary Figure S3. The levels of autophagy and AKT/ERK signaling proteins in U251TR cells**

The U251TR cells were treated with PBS, HOE-642, Ipatasertib (AKT inhibitor), SCH772984 (ERK inhibitor), HOE-642+Ipatasertib, HOE-642+SCH772984, respectively. The relative protein levels of Beclin1, LC3I, LC3II, P62, p-Akt, Akt, p-ERK1/2, ERK1/2 of U251TR cells in each group were analyzed by Western blotting. **P*<0.05, ***P*<0.01.
